# Epidemiology of Endodontic Treatment in Far North Queensland, Australia: A Retrospective Observational Study of 11 Years

**DOI:** 10.1002/cre2.70232

**Published:** 2025-10-14

**Authors:** Aditya Suvarna, Preethi Thennarasu, Sona Sojan, Olivia Gables, Daniel J. Browne, Rodrigo R. Amaral

**Affiliations:** ^1^ College of Medicine and Dentistry James Cook University Cairns Queensland Australia; ^2^ Department of Endodontics College of Dental Medicine, Nova Southeastern University Fort Lauderdale Florida USA

**Keywords:** demographics, endodontic treatment, epidemiology, retrospective study

## Abstract

**Objectives:**

This retrospective observational study assessed demographics, geographical influences, and diagnostic trends in patients receiving endodontic treatment at James Cook University dental clinics between 2011 and 2022. Patient demographics (sex and age), treatment timing, and care types were examined, with a focus on regional and rural disparities in access to endodontic care.

**Material and Methods:**

Data were extracted from electronic dental records from 2011 to 2022 using service item codes under the “Endodontics section (codes 411–459)” of the Australian Schedule of Dental Services and Glossary. The Modified Monash Model framework was used to assess geographical disparities in treatment access.

**Results:**

Females sought treatment earlier than males, who often presented later with more severe symptoms. Older males were more likely to present with pulpal necrosis and chronic apical abscesses, whereas females had higher rates of previously initiated endodontic therapy and asymptomatic apical periodontitis. A total of 2932 patients were treated, with tooth 46 being the most treated (7.54%).

**Conclusions:**

Significant geographical disparities in endodontic treatment timing exist, with rural and remote patients experiencing delays in treatment. These findings highlight the need for improved access to specialized dental care, particularly in underserved areas.

## 1 Introduction

University‐operated dental clinics enable undergraduate dentistry students to develop clinical experience under the guidance of registered and specialist dentists. In Australia, six undergraduate dental clinics exist that facilitate local endodontic care. Understanding the underlying pathobiology of pulpal–periapical diseases is crucial for effective treatment and patient care (Galler et al. 2022; Siqueira and Rôças 2008). This includes a comprehensive understanding of endodontic pathologies and epidemiology, encompassing patient demographics, the distribution of pulp and periapical diseases, the factors influencing their development, and the incidence and prevalence (Shahravan and Haghdoost 2014). It also involves identifying the most commonly affected teeth requiring endodontic treatment (Shahravan and Haghdoost 2014). Additionally, insights into etiological patterns, treatment outcomes, and public health impacts—including the burden of disease, its association with systemic conditions, and economic implications—are crucial for optimizing patient care and preventive strategies (Australian Government 2015; Shahravan and Haghdoost 2014). Such knowledge is vital for tailoring endodontic care to individual needs and guiding workforce planning to reduce the overall burden of endodontic disease (Australian Government 2015).

Globally, advances have been made in understanding endodontic treatment demographics. An estimated 8.2% of teeth worldwide have received endodontic treatment and 55.7% of adults aged 18 years and older have at least one root‐filled tooth (León‐López et al. 2022). Interestingly, the epidemiology of endodontic treatment appears to vary considerably between populations. In a Nigerian cohort, the highest incidence rate of root canal treatment (RCT) (42.7%) occurred between the ages of 20 and 29, the primary indication for RCT was irreversible pulpitis (46.9%), and the most frequently treated teeth were central incisors (Umanah et al. 2014), whereas in a Pakistani cohort, the most common age group (11.3%) was 20–30 years, and the right permanent mandibular first molar was the most frequently treated tooth (Yousuf et al. 2015). A study on Finnish endodontic patients reported the highest incidence of RCT in the 55–59 age group (Vehkalahti et al. 2020). Few studies have explored endodontic demographics in Australia. Da Silva et al. (2009) reported that males aged 50–59 years and females aged 70–79 years were the most common recipients of RCT, with maxillary premolars being the most frequently treated teeth. However, the epidemiology of endodontic treatment in Australia remains largely unassessed, particularly in rural and regional areas, highlighting the need for further research to address gaps in access and quality of endodontic care (Da Silva et al. 2009).

In this study, we sought to investigate the demographics and geographical influences in patients receiving endodontic treatment at the James Cook University (JCU) Dental Clinics in Cairns and Townsville between 2011 and 2022. These clinics are the only undergraduate dental clinics in the 2019 Modified Monash Model (MMM) classification of MMM2 in Queensland. Within Australia, there are seven MMM classifications: Metropolitan Areas (MMM1), Regional Centers (MMM2), Large Rural Towns (MMM3), Medium Rural Towns (MMM4), Small Rural Towns (MMM5), Remote Communities (MMM6), and Very Remote Communities (MMM7) (Australian Government Department of Health, Disability and Ageing 2024). JCU's clinics received patients from all these localities. Additionally, this study sought to investigate the diagnostic trends in endodontic patients. Accurate and complete diagnoses of the pulpal and periapical status of each tooth are critical for determining appropriate treatment plans (Glickman and Schweitzer 2013). Herein, we investigated the rate of recognized pulpal and periapical status of teeth that received RCT (Glickman and Schweitzer 2013). The findings of this retrospective study provided valuable data on the role of university‐operated dental clinics in delivering specialized dental care, particularly in regional and remote areas. Such knowledge is vital for tailoring endodontic care to individual needs, guiding workforce planning, and informing strategies to improve equitable access to endodontic services, ultimately reducing the overall burden of endodontic disease.

## 2 Methods

### 2.1 Ethics

This study was performed according to the principles of the Declaration of Helsinki. Ethics approval for access to dental records was obtained from the university institution's Human Research Ethics Committee (HREC approval no.13368). All patient data were anonymized, and no single‐patient data are identifiable.

### 2.2 Study Participants

**Figure 1:**
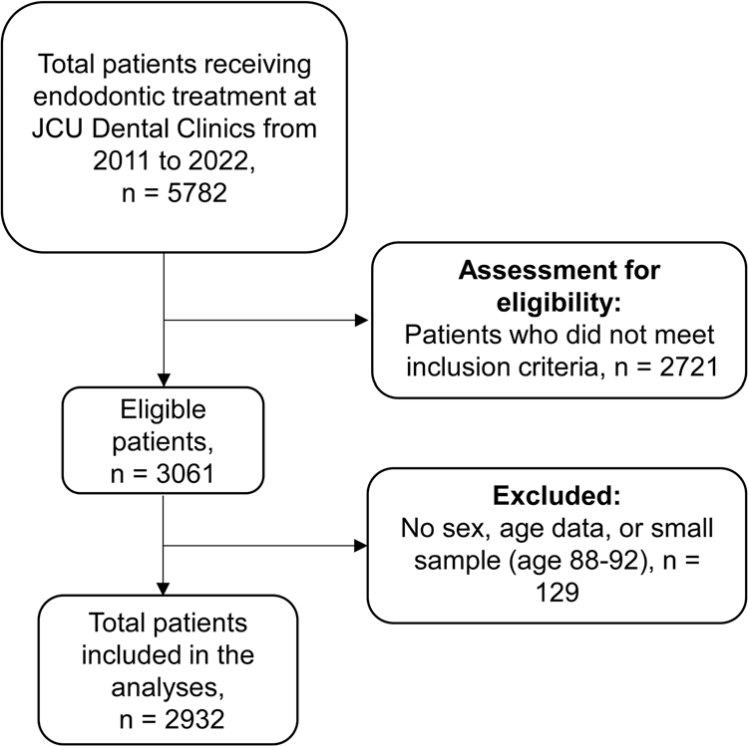
Flow diagram of the inclusion and exclusion criteria for patient inclusion in the study. The inclusion criteria for this study included patients who underwent RCT with a documented reason or diagnosis for commencement. Patients with missing information, such as unstated sex, lack of diagnosis or reason for RCT commencement, and other endodontic procedures such as apicectomy, were excluded.

Patient data were stored on a Titanium Solutions (Auckland, New Zealand) dental management database. For this observational retrospective study, data were extracted from the Titanium® electronic dental records from 2011 to 2022 using service item codes under the “Endodontics section (codes 411–459)” of the Australian Schedule of Dental Services and Glossary. This was done by a Systems Administrator at James Cook University, Cairns, Far North Queensland, Australia. The extracted data were reviewed by the authors for completeness and accuracy, with quality checks including the removal of duplicates and cross‐verification with clinical notes. Data extracted from treatment notes were analyzed and characterized based on patient variables (Table 1). Male and female patients were classified into 5‐year age cohorts, ranging from 18 to 87 years. Patients with unstated age or sex or over 87 years were excluded from the analysis. The patient's residential location was converted into a remoteness area code 1 to 7 using the MMM 2019. As this was a retrospective observational study using all available patient records that fulfilled the inclusion criteria between 2011 and 2022, a formal power calculation was not applicable. The sample size was determined by the number of eligible patients identified during this period, as outlined in the flow diagram (Figure 1).

**Table 1:** Types of extracted data from Titanium® Software.

Variables	Description
Patient demographic	Age
	Sex
	Concession card holder
	Modified Monash Model remoteness of patient residing location
Tooth	Tooth number treated
	Diagnosis and reason for root canal treatment
	Year in which endodontic treatment was commenced
	Completion status of endodontic treatment

### 2.3 Participant Diagnosis

RCT was initiated based on a specific diagnosis or a clinical reason. Herein, we investigated the rate of the current classification of pulpal and periapical diagnoses based on the American Association of Endodontists guidelines (Glickman and Schweitzer 2013). Reasons included cases where RCT was necessary due to factors affecting pulp health or tooth stability, such as extensive caries reaching the pulp and requiring removal, endodontic periodontal lesions, prosthodontic needs, internal or external resorption, or a previous history of dental trauma. Diagnosis, conversely, refers to cases with clinical or radiographic evidence of pathological conditions, such as pulpal necrosis, symptomatic or asymptomatic apical periodontitis, or other definitive signs of pulpal or periapical disease. Diagnosis and reasons were made by students and then confirmed by supervising clinicians. Patients who underwent RCT without a documented reason or diagnosis for commencement were excluded from the study.

### 2.4 Participant Tooth Treated

Participant tooth–treated data were collected from Titanium Solutions' (Auckland, New Zealand) dental management database. The collected data were entered into Microsoft Excel (v16) to generate summaries, including percentages.

### 2.5 Clinic/Study Location

University dental clinics operate as student training facilities equipped with over 100 dental chairs in Cairns and Townsville, in Far North Queensland, Australia. These clinics provide comprehensive dental care to patients across the MMM1‐7 localities.

### 2.6 Statistical Analysis

The data were exported for analysis into the statistical and graphing program, Prism Version 10.2.3 (GraphPad Prism Software). Simple linear regression was applied to explore directional trends in patient numbers across age, sex, diagnosis, and incidence of treatment of tooth, and was chosen for its suitability in assessing large‐scale patterns in stratified observational data. The frequency of endodontic treatment between male and female patients for each tooth, quadrant, and jaw was tested using a Wilcoxon matched‐pairs signed rank test. This nonparametric test was applied to all group comparisons due to the paired nature of male and female comparisons and nonnormal distribution of treatment frequencies. In all cases, unadjusted *p* values < 0.05 were considered statistically significant.

## 3 Results

Throughout 2011–2022, 2932 patients underwent treatment, comprising 1377 males (46.96%) and 1555 females (53.04%). Tooth 46 had the highest incidence of RCT, with 221 patients (7.54%) receiving RCT, followed by Tooth 36, with 207 patients (7.06%). In contrast, Tooth 28 had the lowest incidence, with only two patients (0.07%) undergoing RCT, as detailed in Table 2.

**Table 2:** Distribution of endodontically treated teeth split by sex.

	FDI tooth number	Number of males receiving treatment	Number of females receiving treatment	Total patients receiving treatment
Quadrant 1	11	73 (2.48%)	72 (2.46%)	145 (4.95%)
	12	53 (1.81%)	46 (1.57%)	99 (3.38%)
	13	59 (2.01%)	41 (1.4%)	100 (3.41%)
	14	55 (1.88%)	71 (2.42%)	126 (4.3%)
	15	53 (1.81%)	117 (3.99%)	170 (5.8%)
	16	72 (2.46%)	97 (3.31%)	169 (5.76%)
	17	37 (1.26%)	36 (1.23%)	73 (2.49%)
	18	2 (0.07%)	2 (0.07%)	4 (0.14%)
Quadrant 2	21	68 (2.32%)	60 (2.05%)	128 (4.37%)
	22	51 (1.74%)	48 (1.64%)	99 (3.38%)
	23	48 (1.64%)	33 (1.13%)	81 (2.76%)
	24	48 (1.64%)	81 (2.76%)	129 (4.4%)
	25	61 (2.08%)	105 (3.58%)	166 (5.66%)
	26	65 (2.22%)	97 (3.31%)	162 (5.53%)
	27	31 (1.06%)	36 (1.23%)	67 (2.29%)
	28	1 (0.03%)	1 (0.03%)	2 (0.07%)
Quadrant 3	31	15 (0.51%)	9 (0.31%)	24 (0.82%)
	32	16 (0.55%)	11 (0.38%)	27 (0.92%)
	33	9 (0.31%)	11 (0.38%)	20 (0.68%)
	34	23 (0.78%)	28 (0.95%)	51 (1.74%)
	35	69 (2.35%)	62 (2.11%)	131 (4.47%)
	36	83 (2.83%)	124 (4.23%)	207 (7.06%)
	37	59 (2.01%)	57 (1.94%)	116 (3.96%)
	38	2 (0.07%)	1 (0.03%)	3 (0.1%)
Quadrant 4	41	20 (0.68%)	16 (0.55%)	36 (1.23%)
	42	18 (0.61%)	9 (0.31%)	27 (0.92%)
	43	17 (0.58%)	10 (0.34%)	27 (0.92%)
	44	39 (1.33%)	34 (1.16%)	73 (2.49%)
	45	53 (1.81%)	66 (2.25%)	119 (4.06%)
	46	103 (3.51%)	118 (4.02%)	221 (7.54%)
	47	71 (2.42%)	49 (1.67%)	120 (4.09%)
	48	3 (0.1%)	7 (0.24%)	10 (0.34%)
Total		1377 (46.96%)	1555 (53.04%)	2932 (100%)

### 3.1 Females Receive Endodontic Treatment Earlier in Life Than Males

**Figure 2:**
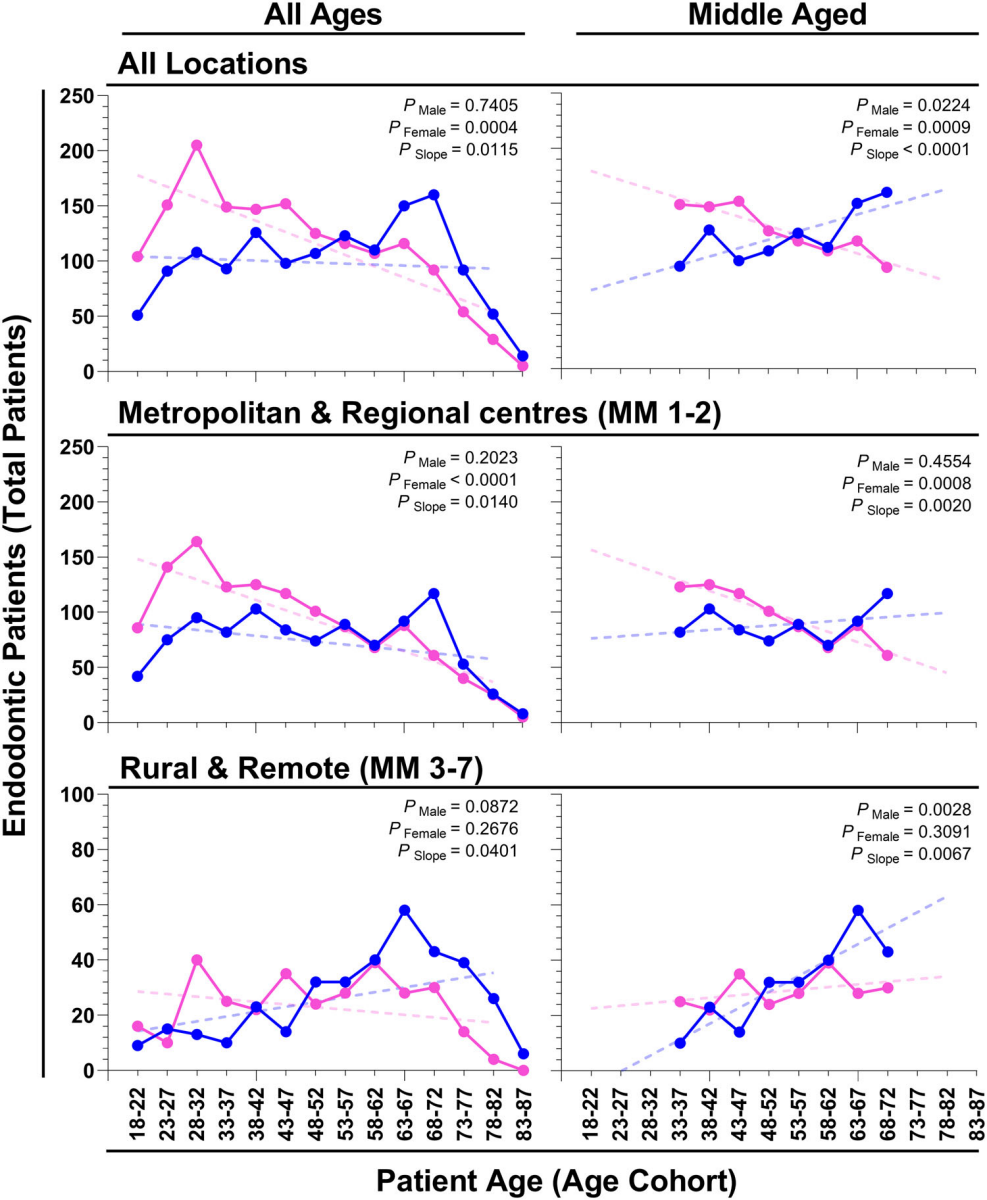
Age, sex, and location of endodontic dental patients treated at James Cook University regional dental clinic from 2010 to 2021. The number of endodontic patients treated in 5‐year age cohorts (Age Cohorts), ranging from 18 to 87 years, is separated by male (blue points) and female (pink points), and categorized by Modified Monash model (MMM) locality (MMM1 to 7). The total number of patients (Total Patients) is characterized either as independent of region (All Regions), from metropolitan and regional centers (MMM1 and MMM2), or from rural and remote (MMM3 to 7) locations. Trending total patient numbers across age cohorts were tested with a linear regression model (dotted line), either considering all patients irrespective of age (All Ages), or considering patients between the ages of 33 and 72 years (Middle‐Aged). Unadjusted *p* values testing either the slope of the line's divergence from 0 for males (*p*_Male_) and females (*p*_Female_), and if the slopes of the male and female lines were different (*p*_Slope_).

To investigate sex‐specific differences in patient demographics, we analyzed the number of male and female patients in 5‐year age cohorts, ranging from 18 to 87 years, over the period 2011 and 2022. The highest number of male patients underwent endodontic treatment between the ages of 68 and 72 years, whereas the highest number of female patients received endodontic care between the ages of 28 and 32 years (All Ages, All locations; Figure 2). Analysis of the trend of sex‐specific patient numbers across all ages found that the number of male patients did not change significantly over time, whereas the number of female patients decreased significantly. The slopes of these trendlines were significantly different (All Ages, All locations: *p*_Male_ = 0.7405, *p*_Female_ = 0.0004, and *p*_Slope_ = 0.0115; Figure 2). When considering younger (Age Cohorts: 18–22, 23–27, and 28–32) and older (Age Cohorts: 73–77, 78–82, and 83–87) endodontic patients, the total patients increased and decreased with age in both sexes, respectively. In contrast, among patients aged 33–72 years, which we defined as “middle‐aged,” the number of male patients significantly increased, whereas the number of female patients significantly decreased and the slopes of these trendlines differed (Middle‐Aged, All locations: *p*_Male_ = 0.0224, *p*_Female_ = 0.0009, *p*_Slope_ < 0.0001; Figure 2). These data indicate age‐related sex‐specific differences in the number of patients receiving endodontic treatment. Specifically, although patient numbers tend to increase early in life and decrease later in life for both sexes, females generally receive endodontic treatment at a younger age than males.

### 3.2 Patients in Metropolitan and Regional Centers Receive Endodontic Treatment Earlier in Life Than Patients From Rural and Remote Locations

To investigate if the sex‐specific differences in the age of receiving endodontic care differed across patient locality, patients were characterized by the MMM (Locations MMM 1 to MMM 7) and grouped as either from metropolitan and regional centers (MMM 1‐2) or from rural and remote locations (MMM 3‐7). Of the 2916 endodontic treatments included in this study, approximately a quarter (675, 23.1%) were patients from rural and remote locations. The majority of patients were from MMM 2 (76.7%), MMM 5 (12.3%), and MMM 4 (9.7%) locations. The highest number of male patients from MMM 1‐2 and MMM 3‐7 underwent endodontic treatment between the ages of 68 and 72 years and 63 and 67 years, respectively, whereas the highest number of female patients received endodontic care between the ages of 28 and 32 years from both MMM 1‐2 and MMM 3‐7 (Figure 2). An analysis of the trend of sex‐specific patient numbers from MMM 1‐2 locations across all ages found that male patient numbers did not change significantly over age, whereas the number of female patients decreased significantly and the slopes of these trendlines differed (All Ages, MMM 1‐2: *p*_Male_ = 0.2023, *p*_Female_ < 0.0001, and *p*_Slope_ = 0.0140; Figure 2). When considering patients from MMM 3‐7 locations across all ages, neither the number of male nor female patients changes significantly over time, although the slopes of these trendlines significantly differed (All Ages, MMM 3‐7: *p*_Male_ = 0.0872, *p*_Female_ = 0.2676, and *p*_Slope_ = 0.0401; Figure 2). These data demonstrate location‐specific differences in the rate of endodontic treatment in females. Specifically, although females from MM1‐2 locations generally receive endodontic treatment earlier in life, females from MM3‐7 locations receive endodontic treatment at a rate with age that more closely matches males.

When considering middle‐aged patients from MMM 1‐2 locations, the number of male patients did not change across age, whereas the number of female patients significantly decreased and the slope of these trendlines differed (Middle‐Aged, MMM 1‐2: *p*_Male_ = 0.4554, *p*_Female_ = 0.0008, *p*_Slope_ = 0.0020; Figure 2). In contrast, the number of male middle‐aged patients from MMM 3‐7 significantly increased with age, whereas the number of female patients remained consistent and the slope of these trendlines differed (Middle‐Aged, MMM 3‐7: *p*_Male_ = 0.0028, *p*_Female_ = 0.3091, *p*_Slope_ = 0.0067; Figure 2). These data demonstrate that the trend toward receiving endodontic treatment later in life was increased in males from MM3‐7 locations. Taken together, these data demonstrate that both male and female patients in metropolitan and regional centers generally receive endodontic treatment earlier in life than patients from rural and remote locations.

### 3.3 Older Females Are More Likely Than Males to be Diagnosed With Asymptomatic Apical Periodontitis

**Figure 3:**
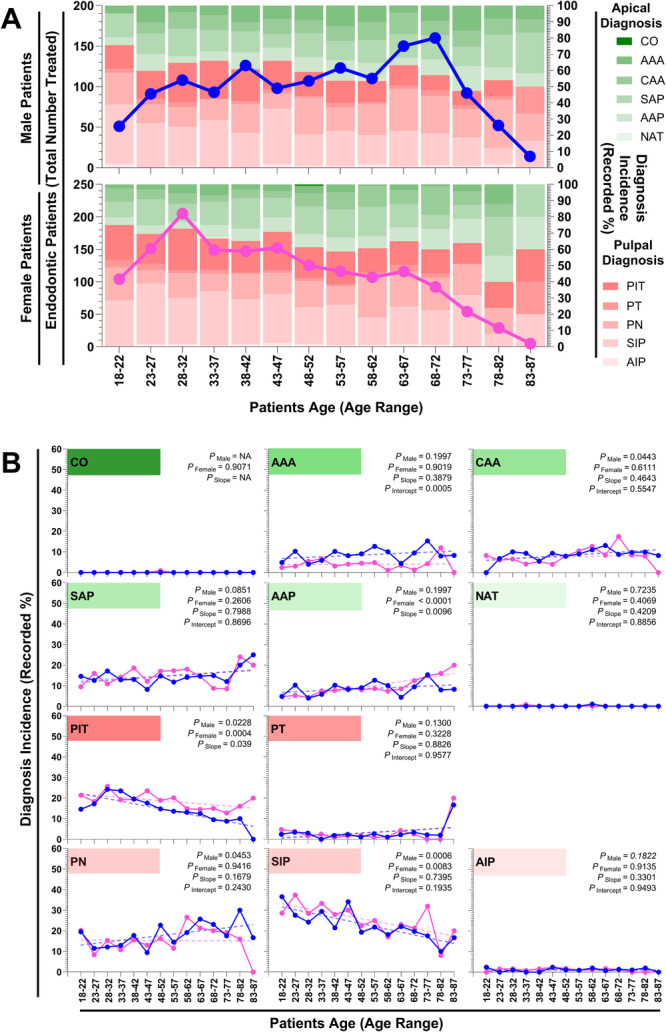
Number and diagnosis of endodontic dental patients treated at James Cook University regional dental clinic from 2011 to 2021. (A) Patient data in 5‐year age cohorts, ranging from 18 to 87 years, are separated by male (blue line) and female (pink line) and characterized by either Pulpal (red bars) or Apical (green bars) diagnoses. Pulpal diagnoses include Asymptomatic Irreversible Pulpitis (AIP), Symptomatic Irreversible Pulpitis (SIP), Pulpal Necrosis (PN), Previously Treated (PT), and Previously Initiated Therapy (PIT). Apical diagnoses include Normal Apical Tissue (NAT), Asymptomatic Apical Periodontitis (AAP), Symptomatic Apical Periodontitis (SAP), Chronic Apical Abscess (CAA), Acute Apical Abscess (AAA), and Condensing Osteitis (CO). (B) The percentage total diagnoses (Recorded %) and a linear regression model (dotted line) of the sex‐specific data are shown. Unadjusted *p* values testing either the slope of the line's divergence from 0 for males (*p*_Male_) and females (*p*_Female_), and if the slope of the male and female lines were different (*p*_Slope_), and if the slope is not different (*p*_Slope_ > 0.05) if the intercept were different (*p*_Intercept_). NA, nonapplicable.

Next, the trends in diagnosis incidence were investigated across age groups and sexes. Specifically, for apical diagnoses (Green Bars; Figure 3), Normal Apical Tissue (NAT) and Condensing Osteitis (CO) were the least common diagnoses. There were no significant differences in the number of NAT diagnoses between sexes, nor in the slopes or intercepts of the male and female trendlines (NAT: *p*_Male_ = 0.7235, *p*_Female_ = 0.4069, *p*_Slopes_ = 0.4209, *p*_Intercept_ = 0.8856; Figure 3). For CO, female diagnoses did not vary with age (*p*_Female_ = 0.4069; Figure 3), and there were no male diagnoses of CO to allow for correlation or trendline analysis. When considering the Asymptomatic Apical Periodontitis diagnosis rate (AAP), males did not vary across age. In contrast, the rate of diagnosis of AAP in females increased significantly with age, and these trendlines differed (AAP: *p*_Male_ = 0.1997, *p*_Female_ < 0.0001, *p*_Slope_ = 0.0096; Figure 3). In contrast, neither male nor female rates of Symptomatic Apical Periodontitis (SAP) diagnosis varied significantly across age, and the trendline and intercepts of these trendlines were not considerably different (SAP: *p*_Male_ = 0.0851, *p*_Female_ = 0.2606, *p*_Slope_ = 0.7988, *p*_Intercept_ = 0.8696; Figure 3). These data suggest that CO, NAT, and SAP apical diagnoses remain consistent across time for both males and females, whereas females are more likely than males to be diagnosed with AAP at older ages.

### 3.4 The Incidence of Chronic Apical Abscess Increases With Age in Males, Whereas Males Are More Likely to be Diagnosed With Acute Apical Abscesses Regardless of Age

When comparing the rates of the apical diagnoses of Chronic Apical Abscess (CAA) or Acute Apical Abscess (AAA) diagnoses, the rates of diagnoses did not change with age in females (*p*_Female_ = 0.6111 and 0.9019 for CAA and AAA, respectively; Figure 3). In males, the rate of AAA diagnosis also remained consistent (AAA: *p*_Male_ = 0.1997; Figure 3); however, the rate of CAA diagnosis significantly increased with age (CAA: *p*_Male_ = 0.0443; Figure 3). Trendline comparisons revealed no significant differences between male and female CAA diagnoses (CAA: *p*_Slope_ = 0.4643, *p*_Intercept_ = 0.5547; Figure 3), and although the slopes of the male and female trendlines of AAA diagnosis were not significantly different, the intercepts were (AAA: *p*_Slopes_ = 0.3879, *p*_Intercept_ = 0.0005; Figure 3). These data indicate that the diagnosis rates of CAA and AAA have a sex‐specific affect. Specifically, although female rates of CAA diagnosis remain consistent, the number of male patients diagnosed with CAA increases with age. Although male and female rates of AAA do not change with age, the rate of AAA diagnosis is consistently higher in males than in females.

### 3.5 Older Males Are More Likely Than Females to Receive a Diagnosis of Pulpal Necrosis and Are Less Likely to Have Previously Initiated Endodontic Therapy

When considering pulpal diagnoses (Red bars; Figure 3), the number of patients diagnosed with Asymptomatic Irreversible Pulpitis (AIP) or Previously Treated (PT) was relatively low (Figure 3). There were no significant differences in AIP diagnoses between sexes across age groups, and the slopes and intercepts of the AIP diagnosis trendlines for males and females were not significantly different (AIP: *p*_Male_ = 0.1822, *p*_Female_ = 0.1822, *p*_Slope_ = 0.3301, *p*_Intercept_ = 0.9493; Figure 3). Similarly, there were no significant differences in PT diagnoses between sexes across age, nor in the slopes or intercept of the PT diagnosis trendlines for males and females (PT: *p*_Male_ = 0.1300, *p*_Female_ = 0.3228, *p*_Slope_ = 0.8826, *p*_Intercept_ = 0.9577; Figure 3). These findings suggest that there is no significant difference in the rates of AIP or PT diagnoses between males and females across age groups. However, there was a spike in PT diagnoses in both male and female patients in the 83–87 age group.

In contrast, a pulpal diagnosis of Symptomatic Irreversible Pulpitis (SIP), Pulpal Necrosis (PN), or Previously Initiated Therapy (PIT) was more common (Figure 3). The number of SIP and PIT diagnoses decreased significantly with increasing age in both males (*p*_Male_ = 0.0006 and 0.0228 for SIP and PIT, respectively; Figure 3) and females (*p*_Female_ = 0.0083 and 0.0228 for SIP and PIT, respectively; Figure 3). Analysis of male and female SIP diagnosis trendlines identified no significant difference in the slopes or intercepts (SID: *p*_Slopes_ = 0.7395, *p*_Intercepts_ = 0.1935; Figure 3), whereas a significant difference was identified between male and female trendline slopes in PIT diagnosis (PIT: *p*_Slopes_ = 0.0390; Figure 3). These data demonstrate that male and female rates of SIP diagnosis are consistent between sexes and consistently decrease with age, whereas PIT diagnosis consistently decreases with age in males and females; fewer males than females are diagnosed with PIT in older age. Similarly, although the slopes and intercepts of the trendlines of male and female diagnoses of PN were not significantly different (PN: *p*_Slopes_ = 0.1679, *p*_Intercepts_ = 0.2430; Figure 3), the number of PN diagnoses of females was consistent over age (PN: *p*_Female *vs*. age_ = 0.9416; Figure 3), and the rate of male PN diagnosis significantly increased (PN: *p*_Male *vs*. age_ = 0.0453; Figure 3). Taken together, these data demonstrate that when receiving a pulpal diagnosis, older males are more likely than females to receive a PN diagnosis and are less likely to receive a PIT diagnosis.

### 3.6 Females Are More Likely Than Males to Have Endodontic Treatment in Maxillary Teeth

**Figure 4:**
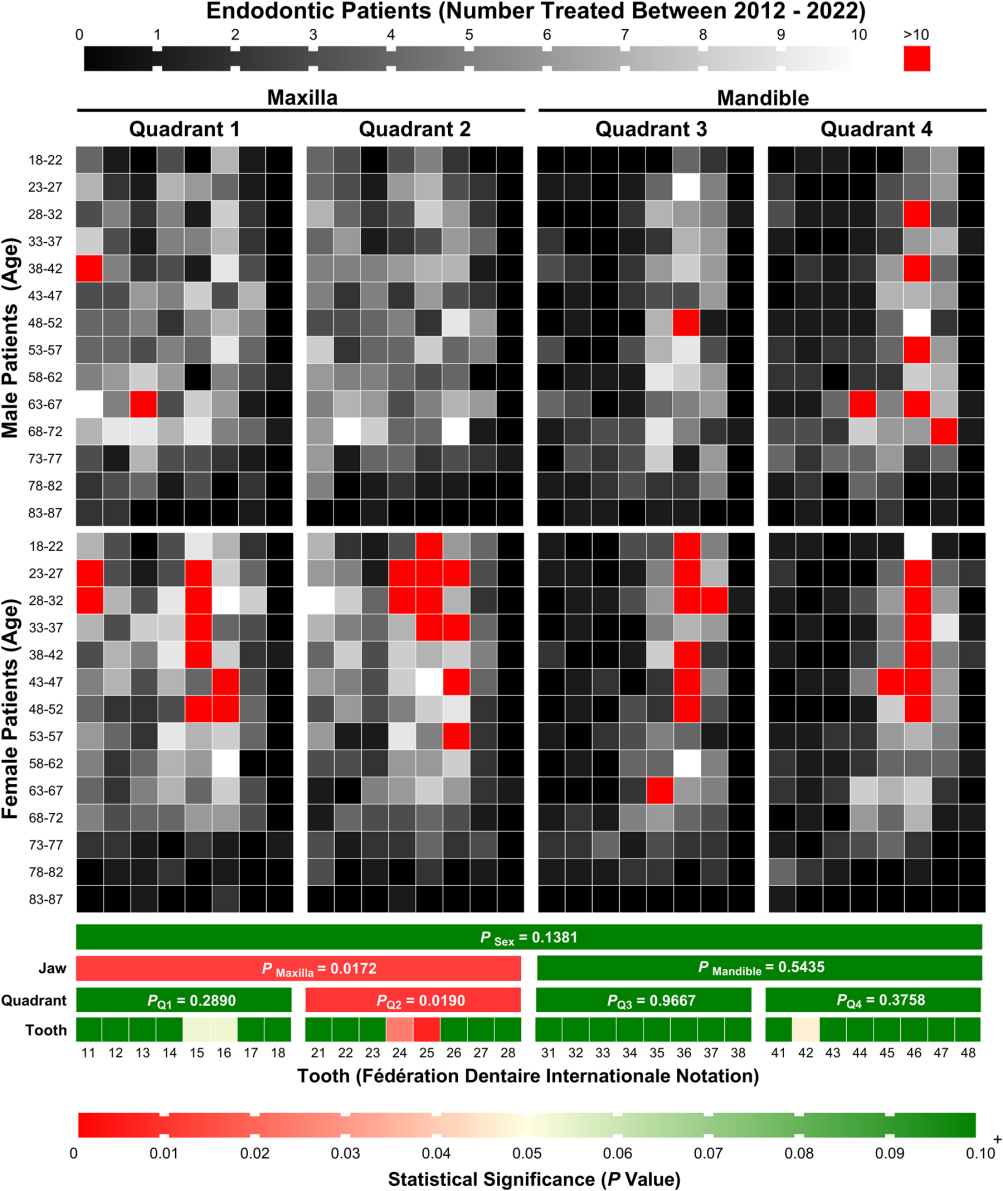
Incidence of endodontic treatment by tooth. The frequency of endodontic treatment for each tooth using Fédération Dentaire Internationale (FDI) notation from 2011 to 2022, separated by sex in 5‐year age groups ranging from 18 to 87 years. The grayscale heatmap indicates the number of treatments (black boxes indicate zero treatments, gray to white boxes are increasing treatments, respectively, and red boxes highlight more than 10 treatments). The incidence of endodontic treatment was compared between all male and female teeth (*p*_Sex_), between the maxilla (*p*_Maxilla_) and the mandible (*p*_Mandible_), across the four quadrants (*p*_Q1_, *p*_Q2_, *p*_Q3_, and *p*_Q4_,), and between individual teeth (Tooth). These comparisons were tested using a Wilcoxon matched‐pairs signed rank test, with statistical significance (*p* value) reported. The red and green heatmaps indicate statistical significance, with selected unadjusted *p* values shown (red boxes indicate statistical significance (*p* < 0.05), whereas green boxes indicate nonsignificant (*p* > 0.05) results).

We next sought to identify if there were sex‐specific differences in the incidence rate of endodontic treatment by tooth when considering all ages. Nonparametric matched‐pairs testing found no significant difference in the incidence rate of endodontic treatment across male and female patients when considering all teeth (*p*_sex_ = 0.1381; Figure 4), teeth in the mandible (*p*_Mandible_ = 0.5435; Figure 4), or teeth in quadrants 1, 3, and 4 (*p*_Q1_ = 0.02890, *p*_Q2_ = 0.9667, and *p*_Q4_ = 0.3758, respectively; Figure 4). In contrast, there was a significant difference between the frequency of endodontic treatment between male and female patients when considering teeth in the maxilla (*p*_Maxilla_ = 0.0172; Figure 4), specifically within quadrant 2 (*p*_Q2_ = 0.0190; Figure 4). When considering individual teeth, females were more likely than males to receive treatment in teeth 24 and 25 (*p*_Tooth_ = 0.0244 and 0.0068, respectively; Figure 4), whereas males received more treatment in tooth 42 (*p*_Tooth_ = 0.0469; Figure 4). These data demonstrate that female patients were more likely to receive endodontic treatment for teeth in the second quadrant of the maxilla.

### 3.7 More Female Teeth Show a Decrease in Endodontic Treatment Incidence With Age Compared With Male Teeth

**Figure 5:**
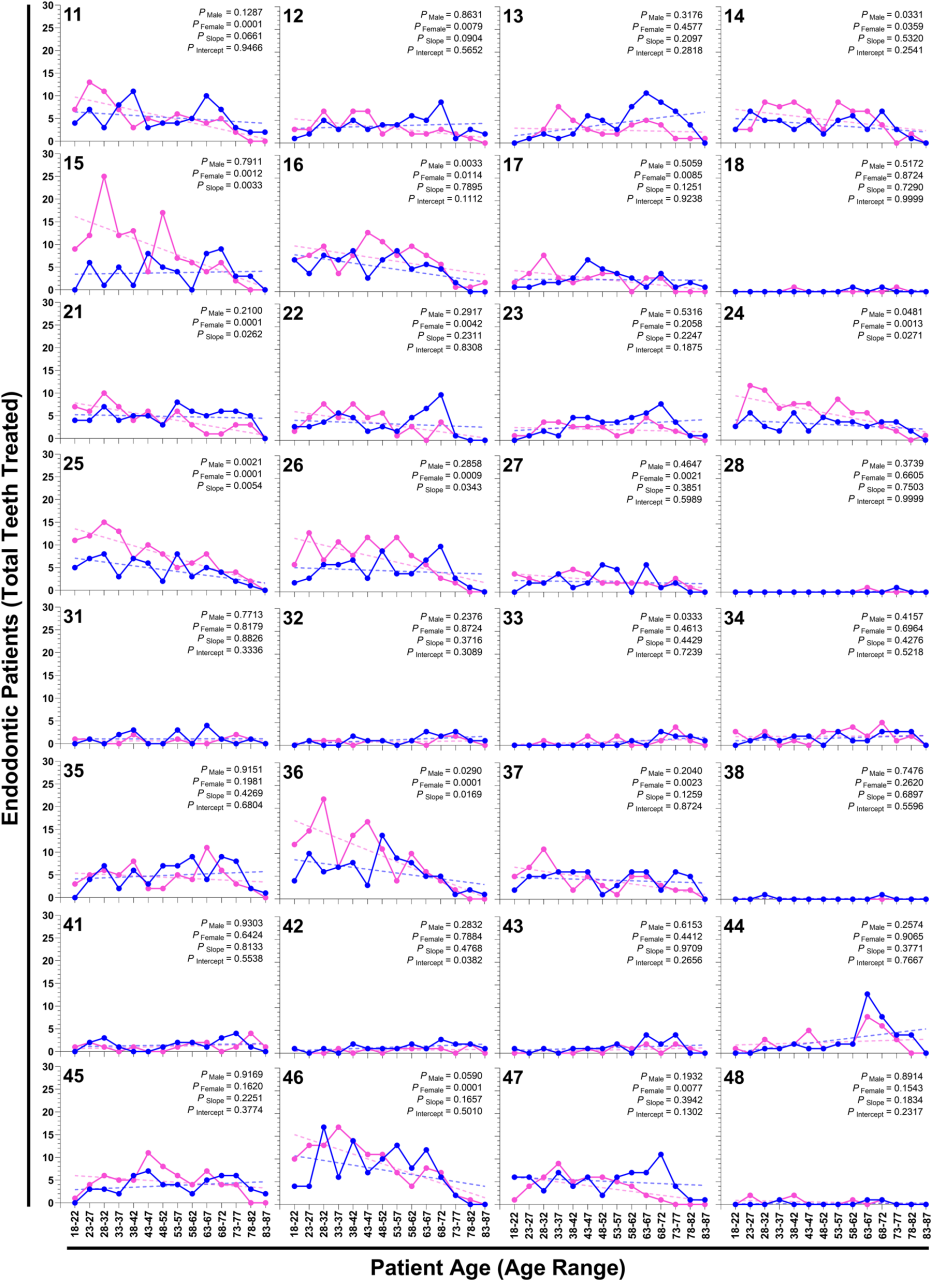
Incidence of endodontic treatment by tooth across patient ages. The frequencies of endodontic treatment for each tooth using Fédération Dentaire Internationale (FDI) notation from 2011 to 2022, separated by sex in 5‐year age groups ranging from 18 to 87 years, are separated by male (blue points) and female (pink points). Trending total endodontic patients (total teeth treated) across age cohorts were tested with a linear regression model (dotted line). Unadjusted *p* values testing either the slope of the line's divergence from 0 for males (*p*_Male_) and females (*p*_Female_), if the slope of the male and female lines were different (*p*_Slope_), and if the slope is not different (*p*_Slope_ > 0.05), if the intercept were different (*p*_Intercept_).

Finally, we sought to investigate if there were sex‐specific differences in the incidence rate of endodontic treatment by tooth across ages. Analysis of the trend of the incidence rate of endodontic treatment significantly varied across age in 18.75% (6/26) of teeth in male patients in tooth 14, 16, 24, 25, 33, and 36 (*p*_Male_ = 0.0331, 0.0033, 0.0481, 0.0021, 0.0333, and 0.0290, respectively; Figure 5). The incidence rate of endodontic treatment varied across age in 50% (16/16) of teeth in female patients in tooth 11, 12, 14, 15, 16, 17, 21, 22, 24, 25, 26, 27, 36, 37, 46, and 47 (*p*_Female_ = 0.0001, 0.0079, 0.0359, 0.0012, 0.0114, 0.0085, 0.0001, 0.0042, 0.0013, 0.0001, 0.0009, 0.0021, 0.0001, 0.0023, 0.0046, and 0.0077, respectively; Figure 5). All teeth that varied significantly in incidence in both male and female patients decreased in treatment incidence over age. When considering the slope of the trendlines between male and female patients, teeth 15, 21, 24, 25, 26, and 36 were significantly different (*p*_Slope_ = 0.0033, 0.0262, 0.0271, 0.0054, 0.0343, and 0.0169, respectively; Figure 5). In all cases, female incidence rates were decreasing more rapidly across age than males. The only intercepts that varied were male and female trendlines in tooth 42 (*p*_Intercept_ = 0.0382; Figure 5), although this tooth was relatively rarely treated (≤ 3 treatments at any age group). Notably, either the highest or the second highest incidence of treatment of teeth 11, 12, 13, 14, 15, 22, 23, 26, 31, 33, 35, 44, and 47 in males occurred between the ages of 68 and 72 years, which produced a late “spike” in the data trendline and aligned with the age when the highest number of male patients received endodontic treatment (Figure 2). These data demonstrate tooth‐specific differences in the incidence rate of endodontic treatment over age in male and female patients. Taken together, the data presented herein demonstrate that over the period of 2011 to 2022, there were age, sex, location, and anatomical differences in the rate of endodontic treatment in patient demographics.

## 4 Discussion

This observational retrospective study analyzed the demographics, geographics, and diagnostic trends of patients receiving endodontic treatment at the JCU Dental Clinics in Cairns and Townsville between 2011 and 2022. Specifically, the study aimed to analyze trends in the timing and type of endodontic care provided and identify disparities in treatment access across metropolitan, regional, and rural areas. To our knowledge, no published literature has assessed the proportion of Australians, particularly in rural and regional areas, who have received RCT. Furthermore, no studies have evaluated patients' demographics, geographic distribution, or diagnostic trends. Consequently, a notable gap exists in our understanding of the epidemiology of endodontic treatment, which differs between geographic locations in Australia, including in Major Cities of Australia, Inner Regional Australia, and Outer Regional Australia. This study seeks to address these gaps by evaluating trends in endodontic care over 11 years at a university‐based dental clinic in regional Queensland.

The findings revealed significant differences in treatment timing between metropolitan/regional (MMM 1‐2) and rural/remote (MMM 3‐7) populations. Specifically, patients from MMM 1‐2 locations received endodontic treatment at younger ages than those in MMM 3‐7 locations, as shown in Figure 2. Although previous studies have highlighted disparities in access to general dental care based on geographic location, this study uniquely applies the MMM classification to demonstrate differences in endodontic treatment timing and demand (Crocombe et al. 2022; Dewanto et al. 2020; Kularatna et al. 2020). No known studies have previously assessed such disparities through the lens of the MMM framework, making this approach valuable for identifying nuanced treatment patterns and access gaps. This discrepancy underscores the impact of geographic and healthcare access disparities on dental care. Limited availability of specialized dental services, fewer practitioners, and logistical challenges in rural areas may delay patient treatment, contributing to the observed trend of later interventions (Melgaço‐Costa et al. 2016; Thanissorn et al. 2023).

Rural populations in Australia face significant oral health disparities, with limited access to care often resulting in delayed treatment and tooth extractions that adversely affect health and well‐being (Emami et al. 2013). Despite growth in the national dental workforce to 65.1 dentists per 100,000 people in 2022, a pronounced maldistribution persists, with 74.9 dentists per 100,000 in urban centers compared with only 20.7 in Remote/Very Remote areas (Sloan et al. 2024). As of September 2024, there were 27,717 registered dental practitioners, including 20,641 dentists, but only 202 endodontists, further restricting specialist access for rural patients (Dental Board AHPRA 2024). These disparities highlight the need for targeted strategies, including mobile services, telehealth, and rural training programs, with the evaluation of the Rural Health Multidisciplinary Training program showing that 75% of graduate dentists in rural Queensland in 2020 came from the JCU program (Barnett et al. 2017; KBC Australia 2020).

The gender distribution in this study highlighted a higher demand for endodontic treatment among females than males, as shown in Figure 2. In particular, females received endodontic care earlier in life, and an increased incidence of RCT completion in the maxilla for females when compared with males was noted. Previous studies have similarly reported higher utilization of endodontic services among women (Albuquerque et al. 2021; Umanah et al. 2014). In addition, the incidence of RCT in younger females has also been noted in various studies (Alkis and Kustarci 2019; Connert et al. 2019; Umanah et al. 2014). These data may indicate greater health awareness and a proactive approach to receiving dental treatment among women. Societal expectations around appearance and health maintenance are speculated to contribute to women's increased utilization of dental services (Albuquerque et al. 2021; Sfeatcu et al. 2022; Umanah et al. 2014). In contrast, Da Silva et al. (2009), in their analysis of patients at Sydney Dental Hospital between June 2006 and June 2008, found higher RCT incidence among males aged 0–39 and 50–69 years, with females surpassing males only in the 40–49 and 70–100 year age groups. However, that study primarily assessed the quality and technical standard of Australian endodontic treatment, rather than patient demographics. Our study highlighted men opting for treatment in the later stages of life. This contrasts with other studies conducted by Hollanda et al. (2008) and De Quadros et al. (2005), which noted a higher prevalence of RCT between the 46–60 age group and the 26–49 age group in males, respectively.

Different healthcare‐seeking behaviors, financial considerations, or perceived needs could influence the observed trend of men opting for endodontic treatment later in life (Hollanda et al. 2008; Sfeatcu et al. 2022). Men may be more likely to defer dental care until conditions become severe, resulting in a higher demand for complex endodontic interventions at older ages (Sfeatcu et al. 2022). This pattern may reflect broader systemic barriers to care, including a lack of education on the importance of preventive oral health measures, socioeconomic constraints, or cultural norms that dissuade men from seeking early treatment (Doğramacı and Rossi‐Fedele 2023; Lipsky et al. 2021; Sfeatcu et al. 2022). Additionally, elderly patients are often more compliant and cooperative; however, significant physical limitations may impede the delivery of dental treatment (Johnstone and Parashos 2015). This could explain the observed decline in the number of patients receiving RCT beyond the age of 68, as found in both male and female cohorts in Figure 3.

Our study identified the permanent mandibular right first molar (FDI notation tooth 46) as the most commonly treated tooth for endodontic procedures at university dental clinics in Table 2. These findings contrast with those reported by Krishnan et al. (2019) and Da Silva et al. (2009), who noted that maxillary premolars were the most frequently treated teeth. However, the study by Krishnan et al. (2019) primarily focused on treatment completion rates rather than patient demographics or geographic trends. This highlights the need for more comprehensive analyses to explore how patient characteristics and treatment settings influence these patterns. Consistent with our results, studies by Ahmed et al. (2009) and Umanah et al. (2014) found that molars were the most endodontically treated tooth. A study conducted by Hollanda et al. (2008) demonstrated a similar pattern to the results from our study, in which premolars and molars were most prevalent. The mandibular first molar holds critical importance in oral health, as it is the first permanent tooth to erupt, typically at approximately 6 years of age, and it remains in the oral cavity for the longest period (Agrawal et al. 2023). This extended exposure significantly increases its susceptibility to dental caries and the likelihood of necessitating endodontic intervention (Agrawal et al. 2023). The pits and fissures of posterior teeth are the most common sites for caries (Agrawal et al. 2023). However, the significant vulnerability of interproximal surfaces highlights the importance of rigorous oral hygiene measures (Agrawal et al. 2023). Practices such as brushing and flossing should be initiated as soon as the first tooth erupts (Agrawal et al. 2023). The early eruption of mandibular first molars further predisposes them to carious lesions, reinforcing the necessity of preventive strategies to mitigate their heightened risk (Agrawal et al. 2023; Ahmed et al. 2009).

The study also examined trends in pulpal diagnoses, revealing notable differences between genders, as shown in Figure 3. Older males were more likely than females to receive a diagnosis of pulpal necrosis and were less likely to have previously initiated endodontic therapy. In addition, the incidence of chronic apical abscess increases with age in males when compared with females. Older males may be less likely to seek dental care due to traditional masculine norms, avoidance behaviors, and limited health literacy. Consequently, they may fail to recognize asymptomatic infections, leading to delayed initiation of endodontic treatment and increased risk of pulpal necrosis (Albuquerque et al. 2021; Sfeatcu et al. 2022; Umanah et al. 2014), whereas females were more likely to have previously initiated endodontic therapy and an asymptomatic apical periodontitis diagnosis, potentially highlighting the contrasted demographic factors when compared with males, as discussed. Studies have found that the frequency of apical periodontitis was higher in root‐filled teeth than in nontreated teeth, proving a potential link to our findings of an increased incidence of asymptomatic apical periodontitis diagnosis as well as a higher rate of previously initiated endodontic therapy in older females when compared with older males (Costa et al. 2019; Tibúrcio‐Machado et al. 2021). This may be due to untreated canals, incomplete instrumentation, or patient factors, leading to persistent bacteria remaining at the apex of the affected teeth (Song et al. 2011).

The study has limitations that should be considered when interpreting the findings. One potential source of bias in this study is the involvement of four different researchers in the data collection and review process. During data collection, researchers had the discretion to determine whether a reason for endodontic treatment was documented. The corresponding data were excluded from the final analysis if no diagnosis was recorded. Additionally, residential postcodes only reflected the patient's current residence rather than their address at the time of treatment, which may impact the interpretation of geographic trends. Furthermore, the inability to provide specific treatment options at a university clinic restricted the scope of care and, consequently, the range of clinical data available. One such example includes vital pulp therapy (VPT), whereby over the 11‐year study period, no cases of VPT were recorded. VPT is a treatment modality aimed pulp vitality, and advances in bioceramic materials have broadened its application to selected cases of irreversible pulpitis where there is no radiographic evidence of apical involvement (Komora et al. 2024; Lin et al. 2020). However, the absence of VPT in this study reflects its exclusion from the treatment protocols at the university during the study period, which restricts both the scope of care and the available clinical data.

The findings of this study are also influenced by the characteristics of patients attending a public university clinic, who may differ in their treatment preferences and willingness to undergo certain procedures compared with patients in private practice. As such, the findings may be more applicable to public dental services or other university‐based clinics, but less representative of private practice settings. Moreover, it should also be acknowledged that university‐affiliated dental clinics may not fully represent the general population or private dental care demographics, which could limit the generalizability of the findings. Additionally, although the patients are given multiple treatment options, ultimately, the decision is influenced by the patients' own preference, which may further affect case selection and outcomes. Finally, as this study examined only one university, the results may not be generalizable to all dental schools or regions. Although this study is limited by limited prior research and excludes treatments performed outside university clinics, the findings highlight sex‐ and geography‐based disparities in endodontic treatment patterns, underscoring the imperative for targeted strategies to improve equitable access and optimize outcomes across diverse patient populations. Future research should investigate demographic variations across multiple university‐affiliated dental clinics in different MMM classifications to better understand disparities in endodontic care.

## 5 Conclusion

Despite its limitations, this study highlights key demographic trends in endodontic care at James Cook University Dental Clinics over an 11‐year period. Females underwent RCT more frequently, at younger ages, and were more likely to receive treatment in maxillary teeth than males. Geographic disparities were evident, with patients from metropolitan and regional areas accessing care earlier than those from rural and remote areas. Distinct age‐ and sex‐related diagnostic patterns were also observed, underscoring the need for multicenter research to determine whether these findings are consistent across Australian university dental clinics.

## Author Contributions

**Aditya Suvarna:** data curation, formal analysis, investigation, writing – original draft, software. **Preethi Thennarasu:** data curation, investigation, writing – original draft, software. **Sona Sojan:** data curation, investigation, writing – original draft, software. **Olivia Gables:** data curation, investigation, writing – original draft, software. **Daniel J. Browne:** data curation, formal analysis, investigation, writing – original draft, software, validation, visualization, writing – review and editing. **Rodrigo R. Amaral:** formal analysis, investigation, methodology, project administration, resources, supervision, validation, visualization, writing – review and editing.

## Conflicts of Interest

The authors declare no conflicts of interest.

## Data Availability

The data that support the findings of this study are available from the corresponding author upon reasonable request.
